# Pharmacodynamic Evaluation of Zoliflodacin Treatment of *Neisseria gonorrhoeae* Strains With Amino Acid Substitutions in the Zoliflodacin Target GyrB Using a Dynamic Hollow Fiber Infection Model

**DOI:** 10.3389/fphar.2022.874176

**Published:** 2022-04-14

**Authors:** Susanne Jacobsson, Daniel Golparian, Joakim Oxelbark, Francois Franceschi, David Brown, Arnold Louie, George Drusano, Magnus Unemo

**Affiliations:** ^1^ WHO Collaborating Centre for Gonorrhoea and Other STIs, National Reference Laboratory for Sexually Transmitted Infections, Department of Laboratory Medicine, Faculty of Medicine and Health, Örebro University, Örebro, Sweden; ^2^ Division of Clinical Chemistry, Department of Laboratory Medicine, Faculty of Medicine and Health, Örebro University, Örebro, Sweden; ^3^ Global Antibiotic Research and Development Partnership (GARDP), Geneva, Switzerland; ^4^ Institute for Therapeutic Innovation, College of Medicine, University of Florida, Gainesville, FL, United States; ^5^ Institute for Global Health, University College London, London, United Kingdom

**Keywords:** *Neisseria gonorrhoeae*, hollow fiber infection model, zoliflodacin, antimicrobial treatment, pharmacodynamics, pharmacokinetics, gyrB, mutant

## Abstract

Novel antimicrobials for effective treatment of uncomplicated gonorrhea are essential, and the first-in-class, oral spiropyrimidinetrione DNA gyrase B inhibitor zoliflodacin appears promising. Using our newly developed Hollow Fiber Infection Model (HFIM), the pharmacodynamics of zoliflodacin was examined. A clinical zoliflodacin-susceptible *N. gonorrhoeae* strain, SE600/18 (harbouring a GyrB S467N amino acid substitution; MIC = 0.25 mg/L), and SE600/18-D429N (zoliflodacin-resistant mutant with a second GyrB substitution, D429N, selected in the HFIM experiments; zoliflodacin MIC = 2 mg/L), were examined. Dose-range experiments, simulating zoliflodacin single oral dose regimens of 0.5, 1, 2, 3, and 4 g, were performed for SE600/18. For SE600/18-D429N, dose-range experiments, simulating zoliflodacin single oral 2, 3, 4, and 6 g doses, and zoliflodacin oral dose-fractionation experiments with 4, 6, and 8 g administered as q12 h were performed. Both strains grew well in the untreated HFIM growth control arms and mostly maintained growth at 10^10^–10^11^ CFU/ml for 7 days. Zoliflodacin 3 and 4 g single dose oral regimens successfully eradicated SE600/18 and no growth was recovered during the 7-days experiments. However, the single oral 0.5, 1, and 2 g doses failed to eradicate SE600/18, and zoliflodacin-resistant populations with a GyrB D429N substitution were selected with all these doses. The zoliflodacin-resistant SE600/18-D429N mutant was not eradicated with any examined treatment regimen. However, this *in vitro*-selected zoliflodacin-resistant mutant was substantially less fit compared to the zoliflodacin-susceptible SE600/18 parent strain. In conclusion, the rare clinical gonococcal strains with GyrB S467N substitution are predisposed to develop zoliflodacin resistance and may require treatment with zoliflodacin ≥3 g. Future development may need to consider the inclusion of diagnostics directed at identifying strains resistant or predisposed to resistance development at a population level and to strengthen surveillance (phenotypically and genetically), and possibly also at the patient level to guide treatment.

## Introduction

The high and increasing levels of antimicrobial resistance (AMR) in *Neisseria gonorrhoeae* globally (Wi et al., 2017; Day et al., 2018; Unemo et al., 2019; Unemo et al., 2021) are seriously threatening the management and control of gonorrhea. The WHO Global Action Plan to Control the Spread and Impact of Antimicrobial Resistance in *N. gonorrhoeae* (WHO, 2012) and WHO Global Action Plan on Antimicrobial Resistance (WHO, 2015) stress that new antimicrobials for treatment of urogenital and extragenital gonorrhea are imperative. Currently, solely two new antimicrobials, zoliflodacin (Jacobsson et al., 2014; Alm et al., 2015; Basarab et al., 2015; Foerster et al., 2015; Unemo et al., 2015; Taylor et al., 2018; Foerster et al., 2019; O’Donnell et al., 2019; Unemo et al., 2019; Bradford et al., 2020; Jacobsson et al., 2021) and gepotidacin (Jacobsson et al., 2018; Scangarella-Oman et al., 2018; Taylor et al., 2018), are in later stages of clinical development for treatment of uncomplicated gonorrhea.

Zoliflodacin is the first-in-class spiropyrimidinetrione and a type II topoisomerase inhibitor with unique target (GyrB) and mode of bactericidal action (Basarab et al., 2015; Kern et al., 2015). Zoliflodacin has a high *in vitro* activity against *N. gonorrhoeae*, including multi-drug-resistant clinical strains (Jacobsson et al., 2014; Unemo et al., 2015; Unemo et al., 2019; Bradford et al., 2020). No clinical *N. gonorrhoeae* isolates with zoliflodacin resistance have been reported when international gonococcal populations from the last decade have been examined (Jacobsson et al., 2014; Unemo et al., 2015; Unemo et al., 2019; Bradford et al., 2020; Le et al., 2021). However, in static *in vitro* laboratory experiments zoliflodacin-resistant mutants have been selected; all containing substitutions of amino acids D429 or K450 of GyrB (Alm et al., 2015; Foerster et al., 2015; Foerster et al., 2019; Jacobsson et al., 2021). No clinical isolate with amino acid substitution in GyrB K450 and only one single clinical isolate with a GyrB D429V substitution has been found (Jacobsson et al., 2014; Alm et al., 2015; Unemo et al., 2015; Unemo et al., 2019; Bradford et al., 2020; Le et al., 2021; Adamson et al., 2021). Additionally, an isolate with a GyrB S467N substitution was selected previously in static *in vitro* experiments (Alm et al., 2015)*.* This substitution did not alone cause zoliflodacin resistance as a first step mutation (zoliflodacin MIC of 0.25 mg/L), however it further increased the MIC of zoliflodacin as a second step mutation (Alm et al., 2015). Rare clinical gonococcal isolates with a GyrB S467N substitution (https://pathogen.watch/collections/all?organismId=485), e.g., 1 of 143 isolates with zoliflodacin MICs of 0.125–0.25 mg/L in Nanjing, China (Le et al., 2021), and wild-type (susceptible) MICs of zoliflodacin have been found. However, the international prevalence of these strains is basically unknown. Overexpression of the MtrC-MtrD-MtrE efflux pump can also further increase the MICs of zoliflodacin (Foerster et al., 2015).

A phase 2 randomised controlled clinical trial (RCT) to evaluate the efficacy and safety of zoliflodacin 2 and 3 g single oral dose for treatment of uncomplicated gonorrhea was recently performed (Taylor et al., 2018). The zoliflodacin 3 g single oral dose was the most effective dose, providing microbiological cure rates of 100% (47/47), 100% (6/6), and 78% (7/9) for urogenital, rectal, and pharyngeal gonorrhea, respectively, in the per protocol analyses. This can be compared to microbiological cure rates of 98% (48/49), 100% (4/4), and 67% (4/6) for urogenital, rectal, and pharyngeal gonorrhea with zoliflodacin 2 g single oral dose. No *N. gonorrhoeae* isolates with *in vitro* resistance to zoliflodacin were found (Taylor et al., 2018). To provide further understanding of the findings of the zoliflodacin phase 2 RCT (Taylor et al., 2018), we developed, optimized and quality assured a dynamic *in vitro* hollow fiber infection model (HFIM) to simulate gonococcal infections and the pharmacokinetic (PK)/pharmacodynamic (PD) of antimicrobials acting against *N. gonorrhoeae* infections (Jacobsson et al., 2021), using geographically, phenotypically and genomically diverse WHO *N. gonorrhoeae* reference strains (Unemo et al., 2015)*.* This HFIM for *N. gonorrhoeae* was used to study the PK/PD of zoliflodacin treatment against *N. gonorrhoeae* strains with full susceptibility to zoliflodacin and no *gyrB* mutations (Jacobsson et al., 2021). Further understanding of the microbiological cures and failures of the treatments in the zoliflodacin phase 2 RCT (Taylor et al., 2018), the zoliflodacin concentration-dependent killing of *N. gonorrhoeae*, and importance of also examining suppression of resistance emergence was provided (Jacobsson et al., 2021). According to the HFIM, for both effective killing and resistance suppression of zoliflodacin wild-type *N. gonorrhoeae* strains, zoliflodacin should be administered at ≥2 g as a single oral dose. However, it was also stated as essential to examine treatment of gonococcal strains with different *gyrB* mutations (Jacobsson et al., 2021).

The main aim of the present study was to examine the pharmacodynamics of zoliflodacin treatment against one clinical zoliflodacin-susceptible *N. gonorrhoeae* strain (SE600/18), with a zoliflodacin-target GyrB S467N substitution, and SE600/18-D429N (zoliflodacin-resistant mutant with an additional *in vitro* selected GyrB D429N substitution) in our dynamic HFIM for *N. gonorrhoeae* (Jacobsson et al., 2021). The biofitness of the zoliflodacin-resistant SE600/18-D429N mutant compared to the zoliflodacin-susceptible clinical SE600/18 strain was also evaluated in the HFIM.

## Materials and Methods

### Bacterial Strains

The clinical zoliflodacin-susceptible *N. gonorrhoeae* strain with GyrB S467N (SE600/18) was cultured in Sweden in 2018. Additionally, the zoliflodacin-resistant mutant of SE600/18 with an additional GyrB D429N substitution selected in the HFIM (SE600/18-D429N) was examined.

### Antimicrobial Susceptibility Testing

For determination of zoliflodacin MICs (mg/L), agar dilution on GCVIT agar plates (Foerster et al., 2019) and microbroth dilution [in triplicates in the HFIM medium, i.e., modified Fastidious Broth (mFB)] were performed, as previously described (Jacobsson et al., 2021). Etest was used to determine MICs (mg/L) of ceftriaxone, cefixime, ciprofloxacin, and azithromycin, in accordance with the manufacturer’s instructions (bioMérieux, Marcy-l’Etoile, France).

### Hollow Fiber Infection Model

For simulation of a gonococcal infection and the PK/PD of current and new antimicrobials, such as zoliflodacin, against *N. gonorrhoeae*, we recently developed and optimized a dynamic HFIM using cellulosic cartridges (FiberCell Systems Inc., Frederick, MD, United States) (Jacobsson et al., 2021)*.* In brief, our HFIM is a two-compartment model system, in which *N. gonorrhoeae* cells grow in the extracapillary space of a cellulosic cartridge containing a bundle of microfibers (FiberCell Systems Inc., Frederick, MD, United States). A syringe pump administered zoliflodacin into the HFIM and peristaltic pumps isovolumetrically replaced zoliflodacin-containing liquid growth medium with zoliflodacin-free liquid growth medium to simulate the half-life of zoliflodacin and non-protein bound (free) zoliflodacin concentration-time profiles reported in human plasma throughout 7 days. *N. gonorrhoeae* quantitative cultures (colony forming units (CFUs)/mL) for total *N. gonorrhoeae* burden and zoliflodacin-resistant *N. gonorrhoeae* population and determination of zoliflodacin concentrations in the HFIM were performed over 7 days (Drusano, 2017).

Briefly, on the first day 0.5 ml of *N. gonorrhoeae* cultures (18–24 h) from GCAGP agar plates (Foerster et al., 2019) were inoculated in 49.5 ml of mFB and incubated at 36°C in a humidified 5% CO_2_-enriched atmosphere to mid-log phase. 10 ml (∼10^6^ CFU/ml) of the *N. gonorrhoeae* suspension were then inoculated into each HFIM cartridge to mimic a clinically relevant *N. gonorrhoeae* cell concentration (Bissessor et al., 2011; Chow et al., 2016; Priest et al., 2017; Van Der Veer et al., 2020). Zoliflodacin was administrated to mimic an adult human PK concentration-time profile following a single oral dose of zoliflodacin [PK parameters for zoliflodacin 3 g oral dose were used (linearly adjusted for other doses): 17% fraction of zoliflodacin free (protein-unbound) in plasma, 6.47 h half-life (t_1/2_), and a 3 h infusion time] (O’Donnell et al., 2019), as previously described (Jacobsson et al., 2021). One HFIM cartridge per examined strain and experiment was used as an untreated growth control.

Dose-range experiments simulated zoliflodacin single dose oral regimens of 0.5, 1, 2, 3, and 4 g against the clinical SE600/18 strain, and single dose oral regimens of 2, 3, 4, and 6 g against SE600/18-D429N. Dose-fractionation experiments simulated zoliflodacin oral dose therapy with 4, 6, and 8 g administered as one half of the total dose given at 0 h and at 12 h (q12 h) against SE600/18-D429N. All experiments were followed for 7 days.

### Quantification of Viable Bacterial Populations

To determine the *N. gonorrhoeae* total population and zoliflodacin-resistant subpopulations, bacterial solution (1 ml) was sampled from each HFIM cartridge at time points 3, 6.5, 24, 48, 72, 96, 120, 144, and 168 h for the dose-range experiments, and at 3, 6.5, 12, 15, 18.5, 24, 48, 72, 96, 120, 144, and 168 h for the q12 h dose-fractionation experiments. Samples were serially diluted in mFB and quantitatively plated on GCAGP agar plates (Foerster et al., 2019) and GCAGP agar plates (Foerster et al., 2019) containing 2 × MIC of zoliflodacin, resulting in a detection limit of ≥100 CFUs per HFIM cartridge, as previously described (Jacobsson et al., 2021). Colony counts (log10 CFU/ml) were quantified after incubation for up to 72 h at 36°C in a humid 5% CO_2_-enriched atmosphere using an automated colony counter (Scan 4000, Interscience, Saint-Nom-la-Bretèche, France).

### Biofitness Experiments

To evaluate the biofitness of the zoliflodacin-resistant mutant selected in the HFIM (SE600/18-D429N) compared to the zoliflodacin-susceptible clinical SE600/18 parent strain, competition experiments using coculture were performed in the HFIM. Briefly, bacteria were harvested from GCAGP agar plates (Foerster et al., 2019) and suspended in mFB to a quantity of ∼10^6^ CFU/ml. Equal volumes (5 ml/strain) of the suspensions of each strain were inoculated into the same HFIM cartridge. Aliquots (1 ml) were sampled at 24, 48, 72, 96, 120, 144, and 168 h, serially diluted in mFB and quantitatively plated on GCAGP agar plates (Foerster et al., 2019), as previously described (Jacobsson et al., 2021). Colony counts (log10 CFU/ml) were quantified after incubation for up to 72 h at 36°C in a humid 5% CO_2_-enriched atmosphere using an automated colony counter (Scan 4000, Interscience, Saint-Nom-la-Bretèche, France). The competitive index (CI) was determined by dividing the ratio of the SE600/18-D429N mutant to wild-type SE600/18 at each time point with the ratio of the SE600/18-D429N mutant to wild-type SE600/18 in the initial inoculum (Vincent et al., 2018).

### Zoliflodacin Concentration Determination

To confirm that the predicted zoliflodacin PK profiles were observed in the HFIM, broth samples (500 µl) were collected at time points 1, 2, 3, 6.5, 18.5, 24, 48, 72, 96, 120, 144, and 168 h for the dose-range experiments, and at 1, 2, 3, 6.5, 12, 15, 18.5, 24, 48, 72, 96, 120, 144, and 168 h for the q12 h dose-fractionation experiments. All zoliflodacin concentrations were determined from 100 μl sample aliquots using liquid chromatography-tandem mass spectrometry (LC-MS/MS), as previously described (Jacobsson et al., 2021).

### Population Pharmacokinetic/Pharmacodynamic Mathematical Modeling

We simultaneously modeled 3 system outputs for the analysis of the experimental data. The system outputs were: concentration of zoliflodacin, total *N. gonorrhoeae* burden, and burden of *N. gonorrhoeae* with lower susceptibility/resistance to zoliflodacin (containing MIC-increasing *gyrB* mutation selected during treatment). Population modeling was performed employing the Non-Parametric Adaptive Grid (NPAG) program of Leary et al. (2001) and Neely et al. (2012). Modeling choices (weighting, etc.) and goodness of fit evaluations were as previously published (Brown et al., 2015). Simulation was performed with the ADAPT V Program of D’Argenio et al. (2009) using Bayesian posterior parameter estimates.

### Comparative Genomic Analysis

Whole genome sequencing (WGS) was performed, as previously described (Jacobsson et al., 2016; Golparian et al., 2020a), on selected colonies that grew on the zoliflodacin-containing plates and that also had increased MICs of zoliflodacin by agar dilution. The WGS was primarily performed to identify zoliflodacin resistance-associated *gyrB* mutations, i.e., the previously identified *gyrB* mutations that were verified to cause the increased MICs of zoliflodacin in *in vitro* selected zoliflodacin-resistant mutants (Alm et al., 2015; Foerster et al., 2015; Foerster et al., 2019; Jacobsson et al., 2021) or novel *gyrB* mutations. However, the whole genome sequences of the zoliflodacin-resistant mutants were examined to identify also any other zoliflodacin resistance-associated mutations selected in the HFIM. All reads were quality controlled and trimmed accordingly using our previously described CLC Genomics Workbench v20.0.4 workflow (Golparian et al., 2020b), and all quality-controlled reads were mapped to the *gyrB* reference obtained from Genbank (Genbank: AE004969.1) using local alignment with CLC Genomics Workbench with match score 1, mismatch cost of 2 and linear gap cost of 3, the variants across the gene were called with a minimum coverage of 10x and a minimum frequency of 35%. WGS reads of SE600/18 with a pre-existing GyrB S467N mutation and the zoliflodacin-resistant mutant of SE600/18 with an additional GyrB D429N substitution (SE600/18-D429N) are available through the European Nucleotide Archive (ENA) accession number PRJEB50904.

The main experiments of the zoliflodacin-susceptible *N. gonorrhoeae* SE600/18 parent strain (with a GyrB S467N mutation) and the zoliflodacin-resistant *N. gonorrhoeae* SE600/18-D429N mutant [with GyrB S467N plus GyrB D429N selected in the Hollow Fiber Infection Model (HFIM)] have been summarized in Supplementary Figure 1.

## Results

### Phenotypic and Genetic Characteristics of Examined *N. gonorrhoeae* Strains

The MICs of zoliflodacin determined using agar dilution and microbroth dilution methods, GyrB substitutions, and additional relevant characteristics of the two examined strains are summarised in Table 1.

**TABLE 1:** Relevant phenotypic and genetic characteristics of *N. gonorrhoeae* strains. Differences between the clinical SE600/18 isolate and the SE600/18-D429N mutant selected in the HFIM are in bold letters.

Strain characteristics	SE600/18	SE600/18-D429N
Zoliflodacin MIC (microbroth MIC)^a^	0.25 (0.5)	**2 (4)**
Ceftriaxone MIC^a^	0.032	0.032
Cefixime MIC^a^	0.125	0.125
Ciprofloxacin MIC^a^	0.5	0.5
Azithromycin MIC^a^	0.125	0.125
Relevant GyrB mutations	S467N	S467N, **D429N**
GyrA codon S91, D95	S91F, D95N	S91F, D95N
*mtrR* promoter region 13 bp inverted repeat	WT	WT
*mtrR* coding region	WT	WT
Mosaic *mtrRCDE*	—	—
PorB1b codon G120, A121	A121D	A121D
NG-MAST	ST20643	ST20643
NG-STAR	ST3537	ST3537
MLST	ST7363	ST7363

MIC, minimum inhibitory concentration; WT, wild type; NA, not applicable; NG-MAST, *N. gonorrhoeae* multiantigen sequence typing; ST, sequence type; NG-STAR, *N. gonorrhoeae* sequence typing antimicrobial resistance; MLST, multi-locus sequence typing.

a MIC (mg/L) was determined using agar dilution and microbroth methods for zoliflodacin, and Etest (bioMérieux, Marcy-l’Etoile, France) for ceftriaxone, cefixime, ciprofloxacin, and azithromycin.

Briefly, the clinical zoliflodacin-susceptible *N. gonorrhoeae* SE600/18 strain, containing a GyrB S467N substitution, and the zoliflodacin-resistant SE600/18-D429N mutant (GyrB S467N plus an *in vitro* selected GyrB D429N substitution) were examined in the HFIM. The zoliflodacin MICs of both strains were one MIC dilution higher using microbroth dilution compared with agar dilution. Notably, SE600/18 belonged to MLST ST7363, which has been a common gonococcal ST internationally during latest decades and it has also been associated with multi-drug resistance, including decreased susceptibility and resistance to extended-spectrum cephalosporins such as ceftriaxone and particularly cefixime (Shimuta et al., 2015; Unemo, 2015; Harris et al., 2018; Sánchez-Busó et al., 2021) (Table 1).

### Hollow Fiber Infection Model results

**FIGURE 1:**
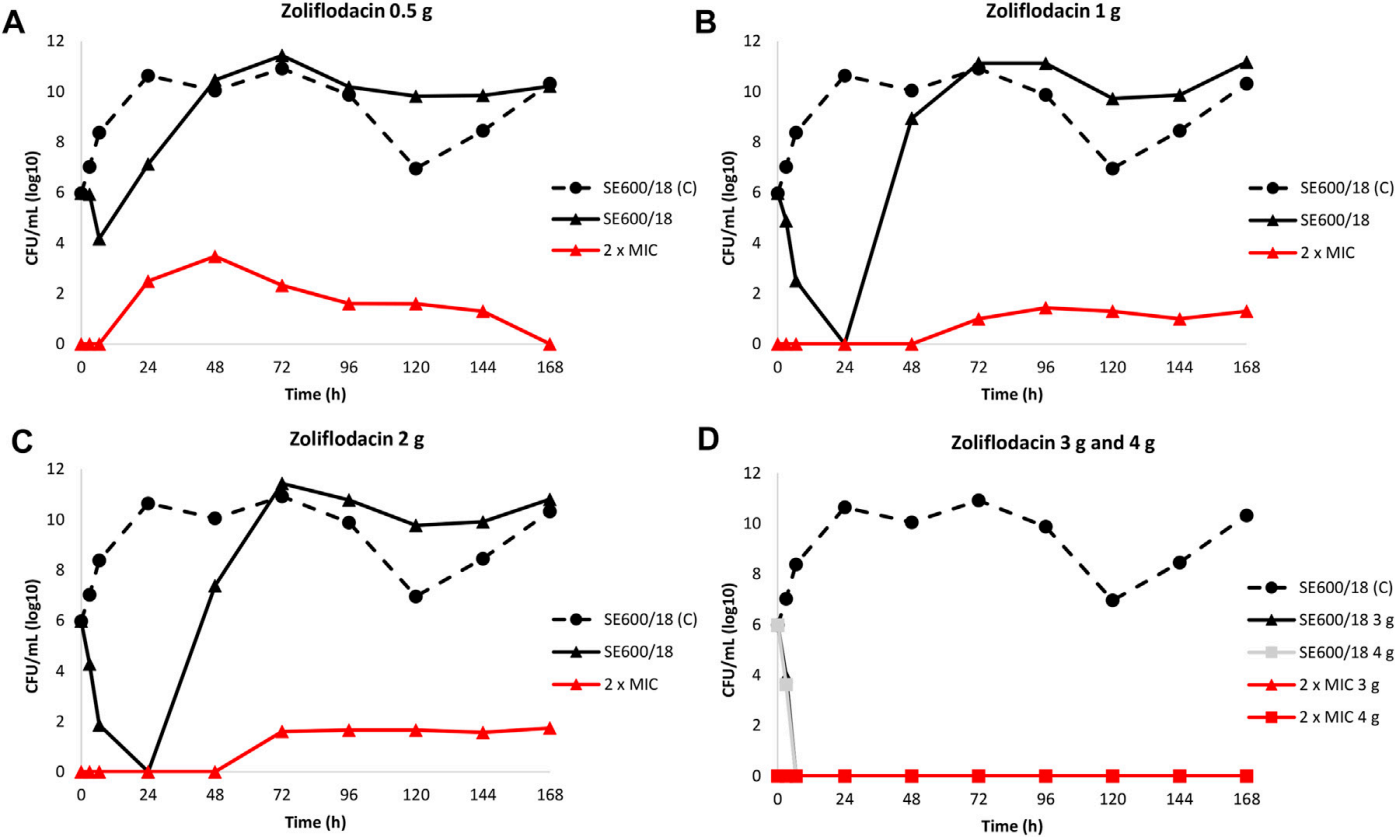
Growth curves of the total population of the clinical zoliflodacin-susceptible *N. gonorrhoeae* strain SE600/18, containing a GyrB S467N amino acid substitution, in the dose-range Hollow Fiber Infection Model experiment simulating zoliflodacin single oral dose of 0.5 **(A)**, 1 **(B)**, 2 **(C)**, 3, and 4 g **(D)** and followed for 7 days are shown (black solid lines). The total growth of zoliflodacin-resistant populations (red lines) on the zoliflodacin-containing plates (2 × MIC) and total growth of the untreated control (C; black dashed line) are also shown for each treatment.

The results of the zoliflodacin dose-range studies for the zoliflodacin-susceptible clinical SE600/18 strain are summarised in Figure 1.

Briefly, the SE600/18 strain grew well in the untreated growth control arms and reached a bacterial density of 10^10^–10^11^ CFU/ml at 24 h (Figure 1). All untreated controls also maintained growth at approximately 10^9^–10^11^ CFU/ml throughout the 7-days experiments (Figure 1). A rapid bacterial kill was documented during the first 6.5 h for all doses. However, after zoliflodacin 0.5 g single dose therapy the SE600/18 strain had recovered growth at 24 h (approximately 10^7^ CFU/ml) and at 48 h the bacterial density was as high as in the untreated control (>10^10^ CFU/ml) (Figure 1A). Using both the zoliflodacin 1 and 2 g treatment (Figures 1B,C, respectively), SE600/18 was rapidly killed and at 24 h no growth was detected. However, at 48 h SE600/18 had recovered growth at bacterial density of approximately 10^9^ CFU/ml (Figure 1B) and 10^7^ CFU/ml (Figure 1C), respectively. Using the zoliflodacin 3 and 4 g treatments, SE600/18 was eradicated at 6.5 h time point and the strain did not recover any growth during the 7-days experiments (Figure 1D).

Zoliflodacin-resistant mutants grew on the zoliflodacin-containing plates for all treatments arms where growth was recovered, i.e., in the 0.5, 1 and 2 g treatment arms (Figures 1A–C). These zoliflodacin-resistant populations emerged after 6.5 h (0.5 g treatment; Figure 1A) or 48 h (1 and 2 g treatments; Figures 1B,C). Notably, these zoliflodacin-resistant populations were maintained at low concentrations of approximately <10^2^–10^4^ CFU/ml during the 7-days experiment. Accordingly, further amplification of the zoliflodacin-resistant populations was not observed, and these zoliflodacin-resistant mutant populations appeared to grow slower and in smaller colonies compared to the SE600/18 strain on agar plates, which all may indicate a suboptimal biofitness. These selected zoliflodacin-resistant mutants had zoliflodacin MICs of 2 mg/L (agar dilution) and contained the GyrB D429N substitution in addition to the pre-existing GyrB S467N substitution (referred to as SE600/18-D429N hereafter). No other selected mutations that appeared to be associated with zoliflodacin susceptibility were found.

**FIGURE 2:**
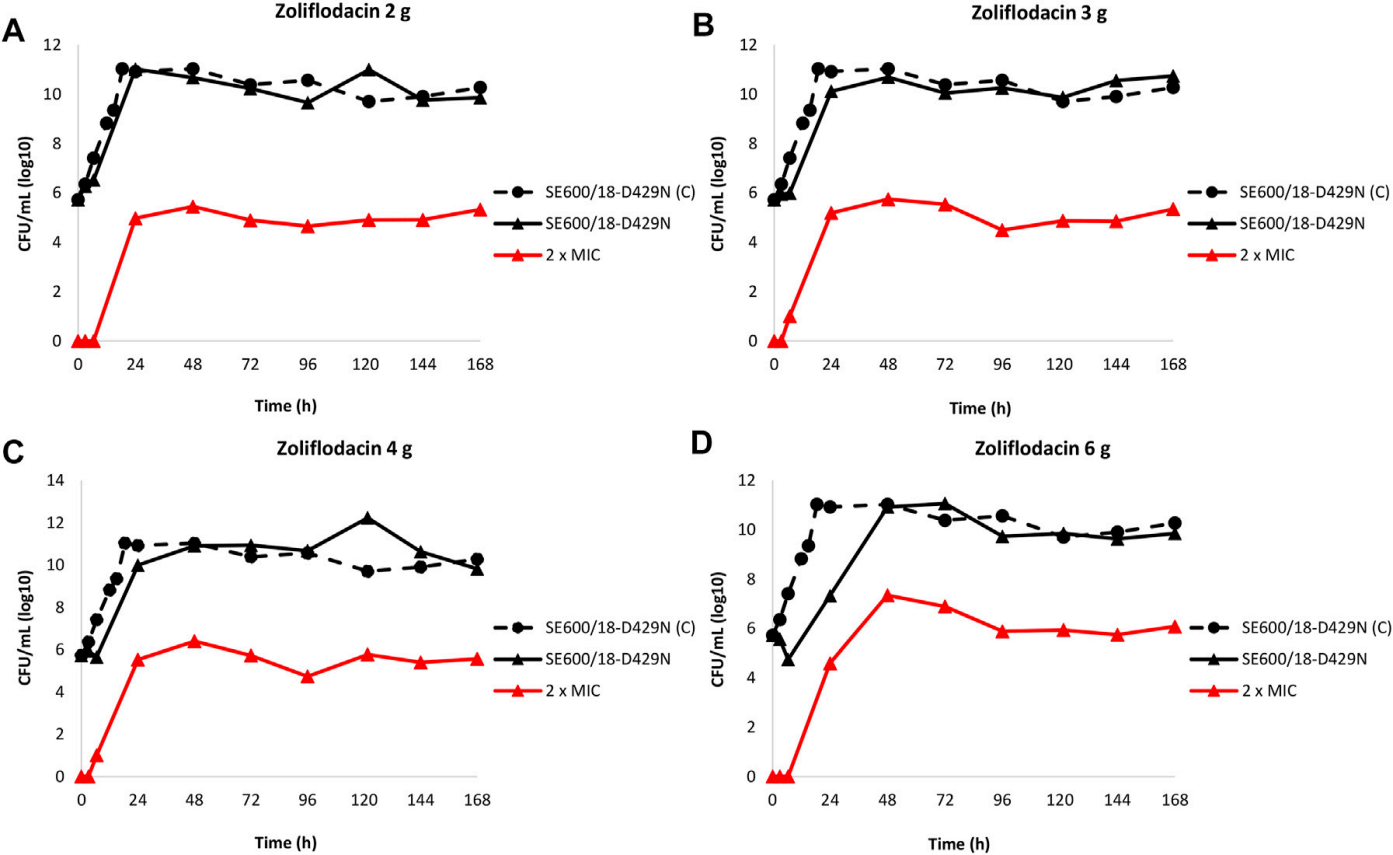
Growth curves of the total population of the zoliflodacin-resistant *Neisseria gonorrhoeae* SE600/18-D429N mutant (containing GyrB S467N amino acid substitution plus an *in vitro* selected D429N substitution), in the dose-range Hollow Fiber Infection Model experiment simulating zoliflodacin single oral dose of 2 **(A)**, 3 **(B)**, 4 **(C)**, and 6 g **(D)** and followed for 7 days are shown (black solid lines). The total growth of population with increased resistance (red lines) on the zoliflodacin-containing plates (2 × MIC) and total growth of the untreated control (C; black dashed line) are also shown for each treatment.

The results of the zoliflodacin dose-range studies for the zoliflodacin-resistant SE600/18-D429N mutant are summarised in Figure 2.

In brief, the SE600/18-D429N mutant grew well in the untreated growth control arms and reached a bacterial density of 10^11^ CFU/ml at 24 h (Figure 2). All untreated controls maintained growth at around 10^10^–10^11^ CFU/ml throughout the 7-day experiments (Figure 2). However, the zoliflodacin 2, 3, and 4 g single dose oral therapy did not result in any kill of SE600/18-D429N (Figures 2A–C) and the zoliflodacin 6 g single dose regimen only caused bacterial kill the first 6.5 h (Figure 2D). Growth was recovered in all treatment arms and ranged from approximately 10^7^ CFU/ml (6 g arm) to 10^11^ CFU/ml (2 g arm) at 24 h and at the bacterial density of the untreated control at 48 h (10^11^ CFU/ml), which was maintained during the remaining 5 days of the experiments (Figure 2).

Furthermore, zoliflodacin-resistant gonococcal populations grew on the zoliflodacin-containing plates for all treatments arms (Figures 2A–D). These zoliflodacin-resistant populations grew at approximately 10^5^ CFU/ml after 24 h in all treatment arms. Notably, these zoliflodacin-resistant populations fluctuated at 10^5^–10^7^ CFU/ml during the whole 7-days experiment (Figure 2). Accordingly, further amplification of these zoliflodacin-resistant populations was not observed, and these mutant populations appeared to grow slower and in smaller colonies on agar plates, which all may indicate a suboptimal biofitness. These zoliflodacin-resistant populations had zoliflodacin MICs of 2–8 mg/L (agar dilution) and contained the GyrB D429N substitution in addition to the pre-existing GyrB S467N substitution.

**FIGURE 3:**
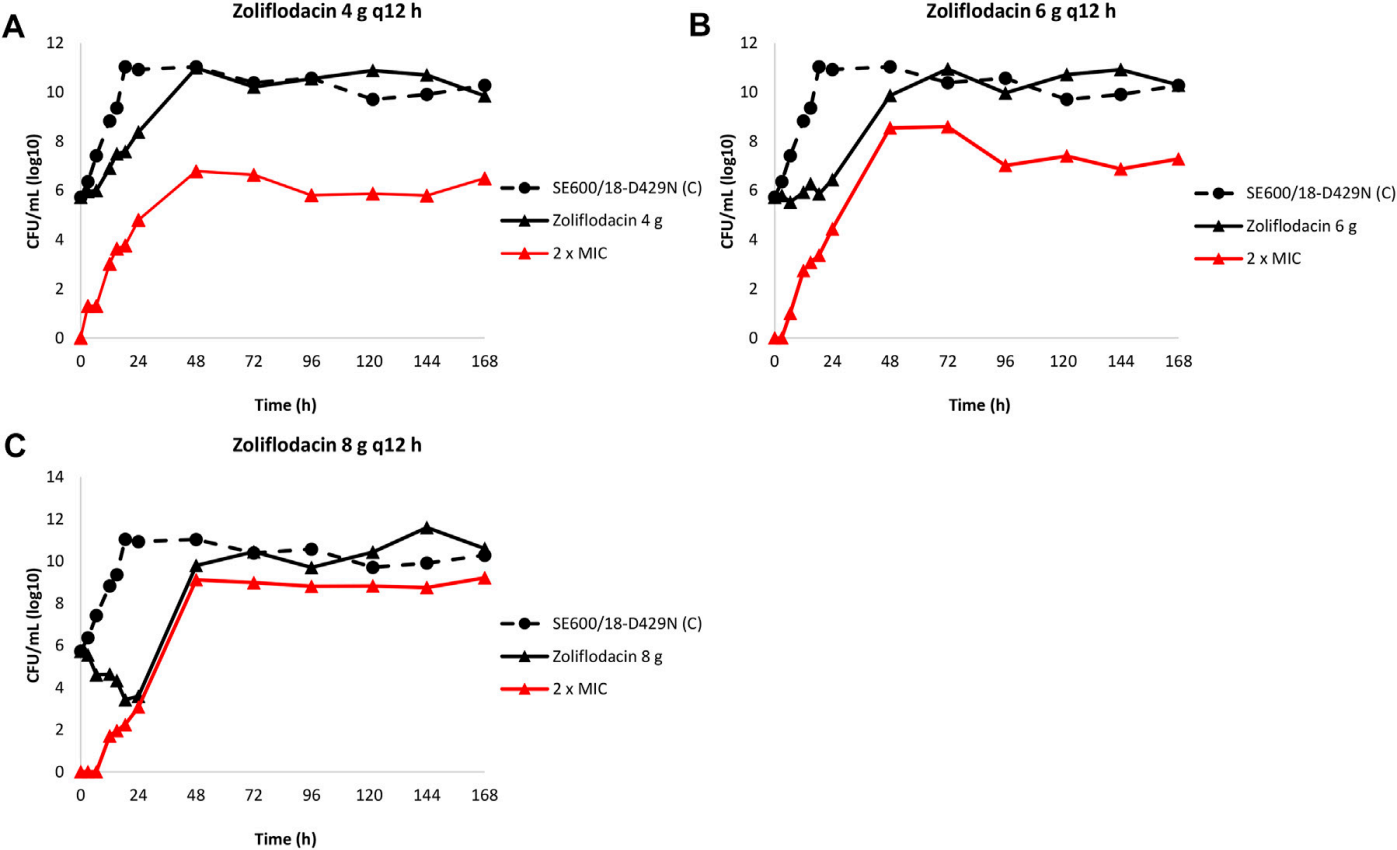
Growth curves of the total population of the zoliflodacin-resistant *Neisseria gonorrhoeae* SE600/18-D429N mutant (containing the GyrB S467N amino acid substitution plus an *in vitro* selected D429N substitution), in the dose-range Hollow Fiber Infection Model experiment simulating a zoliflodacin oral fractionated dose of 4 g (2 g given at 0 and 12 h) **(A)**, 6 g (3 g given at 0 and 12 h) **(B)**, and 8 g (4 g given at 0 and 12 h) **(C)** over 24 h and followed for 7 days are shown (black solid lines). The total growth of population with increased resistance (red lines) on the zoliflodacin-containing plates (2 × MIC) and total growth of the untreated control (C; black dashed line) are also shown for each treatment.

The results of the zoliflodacin dose-fractionation experiments for the zoliflodacin-resistant SE600/18-D429N mutant are summarised in Figure 3.

Briefly, the SE600/18-D429N mutant grew well in the untreated growth control arms and reached a bacterial density of 10^11^ CFU/ml at 24 h (Figure 3). All untreated controls maintained growth at around 10^10^–10^11^ CFU/ml throughout the 7-day experiments (Figure 3). The equivalent zoliflodacin oral therapy of 4 and 6 g administered as equally divided doses at q12 h did not result in any substantial kill of SE600/18-D429N (Figures 3A,B) and the zoliflodacin 8 g q12 h regimen only caused bacterial kill the first 24 h (Figure 3C). Accordingly, growth was recovered in all treatment arms and ranged from approximately 10^3^ CFU/ml (8 g q12 h arm) to 10^8^ CFU/ml (4 g q12 h arm) at 24 h and the bacterial density at 48 h was at approximately 10^10^–10^11^ CFU/ml, which was maintained during the remaining 5 days of the experiments (Figure 3).

Zoliflodacin-resistant populations grew on the zoliflodacin-containing plates for all treatments arms (Figures 3A–C). These zoliflodacin-resistant populations grew at approximately 10^3^–10^4^ CFU/ml after 24 h and at >10^6^–10^9^ CFU/ml at 48 h in all treatment arms (Figure 3). The zoliflodacin-resistant population selected in the zoliflodacin 8 g q12 h treatment arm appeared to recover growth at nearly the same bacterial density as the untreated control (Figure 3C), however, also this mutant population appeared to grow slower and in smaller colonies compared to the SE600/18 strain on agar plates, which may indicate a suboptimal biofitness. These zoliflodacin-resistant populations had zoliflodacin MICs of 2–8 mg/L (agar dilution) and contained the GyrB D429N substitution in addition to the pre-existing GyrB S467N substitution.

### Population Pharmacokinetic/Pharmacodynamic Modeling

The three output PK/PD model was fit to all the data for SE600/18. The mean and median values for SE600/18 are displayed in Table 2.

**TABLE 2:** Mean, median and standard deviation of the parameter values for the Hollow Fiber Infection Model study with the *N. gonorrhoeae* clinical SE600/18 strain with a pre-existing GyrB S467N amino acid substitution.

Parameter	Mean	Median	Standard deviation
V_c_ (L)	1073	99.03	1050
CL (L/hr)	116.3	13.11	107.2
K_g-s_ (hr^−1^)	0.68	0.117	0.591
K_g-r_ (hr^−1^)	0.088	0.066	0.050
K_kill-s_ (hr^−1^)	9.087	5.218	4.065
K_kill-r_ (hr^−1^)	1.820	0.520	1.759
C_50-s_ (mg/L)	0.724	0.134	0.670
C_50-r_ (mg/L)	2.527	1.274	1.802
H_s_ (—)	4.202	3.191	2.350
H_r_ (—)	13.86	5.663	12.78
POPMAX (CFU/ml)	0.261 × 10^11^	0.109 × 1011	0.337 × 10^11^
IC2 (CFU/ml)	8.789 × 10^5^	1.066 × 105	9.209 × 10^5^
IC3 (CFU/ml)	5.012	3.059	3.769

V_c_, apparent volume of the central compartment; CL, clearance; K_g-s_ and K_g-r_, rate constants of growth for the susceptible and resistant population, respectively; K_kill-s_ and K_kill-r_, rate constants of kill for the susceptible and resistant population, respectively; C_50-s_ and C_50-r_, concentrations of zoliflodacin at which the kill rate is half maximal for the susceptible and resistant population, respectively; H_s_ and H_r_, Hill’s constants for the susceptible and resistant populations, respectively (unitless); POPMAX, maximal population size; CFU, colony forming units; IC_2_ and IC_3_, sizes of the total and resistant populations, respectively, at therapy initiation.

The fit of the model to the data was acceptable, with exception of the pre-Bayesian (population) analysis for the zoliflodacin-resistant mutants (SE600/18-D429N). The predicted-observed regressions for the analysis are displayed in Supplementary Figure 2. The reason for the poor model fit for the zoliflodacin-resistant mutants is likely because of poor biofitness of these zoliflodacin-resistant mutants. This hypothesis is also supported by their very small K_g-r_ (Table 2) and, even when zoliflodacin was essentially gone, the zoliflodacin-resistant mutants did not amplify (Supplementary Figure 2; Figure 1).

Regarding the zoliflodacin PK profiles, the agreement between observed and predicted zoliflodacin concentrations in the HFIM during the experiments were high (Supplementary Figures 2A,D).

For the HFIM dose-ranging study for the clinical *N. gonorrhoeae* strain SE600/18, we performed simulation with the identified parameter values to obtain an approximate exposure of zoliflodacin that would suppress amplification of selected mutants with increased zoliflodacin MIC and *gyrB* resistance mutations. That exposure was a zoliflodacin 2.7 g single dose oral treatment in the HFIM.

The growth rate constant for the susceptible SE600/18 population (Table 2) was 58–60% of the growth rate constants previously measured for WHO F and WHO X (Jacobsson et al., 2021). However, the growth rate constant for the zoliflodacin-resistant SE600/18-D429N population (Table 2) was only 7–16% of the corresponding growth rate constants for zoliflodacin-resistant populations of WHO F and WHO X (Jacobsson et al., 2021). This suboptimal growth rate of the zoliflodacin-resistant SE600/18-D429N population shows that its growth is impaired and accordingly biofitness decreased, which was further supported by the kill rate constant for the zoliflodacin-resistant SE600/18-D429N population that was more than 20 times higher than the growth rate constant for the mutant (Table 2).

### Competition Biofitness Experiments Using Coculture in the HFIM

**FIGURE 4:**
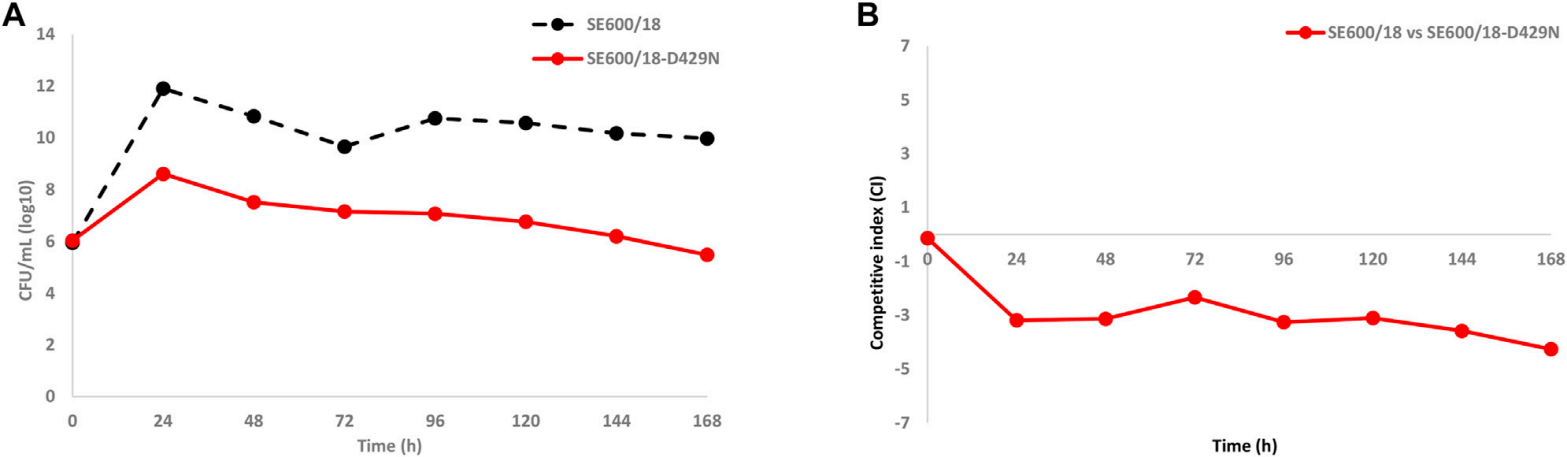
**(A)** Growth curves of the total population of the zoliflodacin-susceptible clinical *Neisseria gonorrhoeae* SE600/18 strain (black dashed line) and the zoliflodacin-resistant *N. gonorrhoeae* SE600/18-D429N mutant (containing the pre-existing GyrB S467N amino acid substitution plus an *in vitro* selected D429N substitution; red solid line), when cocultured in the same Hollow Fiber Infection Model (HFIM) cartridge and followed for 7 days. **(B)** Competitive indexes for the zoliflodacin-susceptible clinical *Neisseria gonorrhoeae* SE600/18 strain and the outcompeted zoliflodacin-resistant *N. gonorrhoeae* SE600/18-D429N mutant (red solid line), when cocultured in the same HFIM cartridge and followed for 7 days.

To confirm if the *in vitro*-selected zoliflodacin resistance in the clinical zoliflodacin-susceptible SE600/18 strain impaired bacterial growth and accordingly decreased the biofitness, the zoliflodacin-susceptible parent SE600/18 and the zoliflodacin-resistant SE600/18-D429N mutant were cocultured in the same HFIM cartridge for 7 days (Figure 4A) and the competitive index calculated (Figure 4B). The growth of the zoliflodacin-susceptible clinical SE600/18 strain was maintained at ∼10^10^–10^12^ CFU/ml during the 7 days experiments, which was at a similar level as in the SE600/18 monocultures. However, the growth of the zoliflodacin-resistant SE600/18-D429N mutant was substantially lower particularly during the first 24 h and it peaked at ∼10^8^ CFU/ml at 24 h and then decreased for each day resulting in a bacterial density of ∼10^6^ CFU/ml after 7 days (Figure 4A). The clinical zoliflodacin-susceptible SE600/18 strain appeared to outcompete the zoliflodacin-resistant SE600/18-D429N mutant, which was even more clearly shown when plotting the competitive index over the 7 days experiments (Figure 4B). Accordingly, the *in vitro*-selected zoliflodacin-resistant mutant SE600/18-D429N was substantially less fit compared to the zoliflodacin-susceptible clinical SE600/18 parent strain (Figure 4).

## Discussion

The high levels of AMR in *N. gonorrhoeae* globally (Wi et al., 2017; Day et al., 2018; Unemo et al., 2019; Unemo et al., 2021) is seriously threatening the management and control of gonorrhea, and novel antimicrobials for effective treatment of urogenital and extragenital gonorrhea are urgently needed. The novel spiropyrimidinetrione zoliflodacin has been shown to be effective in treating gonococcal urogenital and rectal infections (Taylor et al., 2018). A single oral zoliflodacin 3 g dose was shown to cure all anogenital gonococcal infections and most (78%, 7/9) of the included pharyngeal infections, and no isolates with zoliflodacin resistance were found (Taylor et al., 2018). Recently, using our newly developed dynamic HFIM for *N. gonorrhoeae*, we verified that zoliflodacin administered as single oral doses ≥2 g is sufficient to eradicate infections caused by zoliflodacin-susceptible *N. gonorrhoeae* strains (wild-type *gyrB* gene and zoliflodacin MICs (0.064–0.125 mg/L)), which were effectively killed while also supressing resistance to zoliflodacin (Jacobsson et al., 2021). However, it was additionally stated as essential to examine treatment of *N. gonorrhoeae* strains with relevant *gyrB* mutations (Jacobsson et al., 2021).

In the present study, we examined the clinical zoliflodacin-susceptible *N. gonorrhoeae* strain SE600/18 with a pre-existing GyrB S467N substitution (zoliflodacin MIC = 0.25 mg/L in agar dilution). When treating SE600/18 with zoliflodacin single oral dose of 0.5–4 g in the HFIM, the SE600/18 strain was initially rapidly killed, however, with zoliflodacin 0.5, 1, and 2 g single oral doses the strain had recovered growth at 24 h (zoliflodacin 0.5 g) or at 48 h (zoliflodacin 1 and 2 g). Furthermore, zoliflodacin-resistant populations started to amplify after 6.5 h (zoliflodacin 0.5 g) or after 48 h (zoliflodacin 1 and 2 g). The zoliflodacin-resistant mutants of SE600/18 all contained an additional GyrB substitution (SE600/18-D429N; resulting in zoliflodacin MIC = 2 mg/L in agar dilution) (Alm et al., 2015; Foerster et al., 2015; Foerster et al., 2019; Jacobsson et al., 2021). Accordingly, zoliflodacin 2 g single oral dose failed to eradicate SE600/18 in the HFIM, which further strengthens previous evidence that single oral dose of zoliflodacin >2 g can be required for effective treatment of rare gonococcal strains (Taylor et al., 2018; Jacobsson et al., 2021), e.g., strains with the GyrB S467N substitution (require ≥2.7 g according to our PK/PD modeling). Our gonorrhea treatment simulations in the HFIM also showed that zoliflodacin-resistant mutants with GyrB S467N plus D429N substitution, if selected by suboptimal zoliflodacin exposures, may not be effectively treated with zoliflodacin single oral dose of 2–6 g or 4–8 g q12 h. Additionally, our HFIM results suggest that zoliflodacin-resistant mutants are selected at a higher frequency with zoliflodacin doses ≤2 g when a strain has the pre-existing GyrB S467N substitution, i.e., compared to the previously examined zoliflodacin-susceptible *N. gonorrhoeae* reference strains WHO F and WHO X with wild type *gyrB* gene (Jacobsson et al., 2021). Thus, the GyrB S467N substitution appears to predispose to emergence of zoliflodacin resistance, despite not conferring resistance to zoliflodacin on its own. Fortunately, *N. gonorrhoeae* strains with GyrB S467N substitution appear to be very rare internationally (https://pathogen.watch/collections/all?organismId=485; Le et al., 2021). Furthermore, the zoliflodacin-resistant SE600/18-D429N mutant suffered from a biofitness disadvantage and was outcompeted by the zoliflodacin-susceptible parent SE600/18 strain, which suggests that these zoliflodacin-resistant strains will be less effective at amplifying and spreading after emergence.

It is important to continue to survey phenotypic zoliflodacin susceptibility, but our data demonstrate the need to also consider the surveillance of known *gyrB* resistance mutations (in amino acid codons for D429 and K450), the GyrB S467N substitution and other mutations in *gyrB* or other genes that potentially cause resistance to zoliflodacin or could predispose for zoliflodacin resistance emergence. The failure to eradicate a *N. gonorrhoeae* GyrB S467N strain with up to zoliflodacin 2 g single oral dose (≥2.7 g required according to our PK/PD modeling) in this study, the higher selection rate of GyrB D429N resistance mutations in the GyrB S467N strains, and the underlying human inter-population PK variance could suggest that 100% of gonococcal infections caused by the sporadic gonococcal GyrB S467N strains may not be eradicated with a single oral dose of 3 g zoliflodacin, at all body sites.

The limitations of this study include the absence of zoliflodacin PK data from the infection sites for gonorrhea, such as the anogenital tract and the oropharynx. Consequently, the HFIM gonorrhea treatment simulations had to be based on concentrations of free zoliflodacin in human plasma which may not ideally reflect the urogenital and extragenital infection sites. Nevertheless, human plasma antimicrobial concentrations are commonly used as surrogates for concentrations of the antimicrobials at the infection sites for many bacterial infections (due to the lack of measured infection site concentrations), and mostly, these surrogates are sufficient to link drug exposure to effect (Drusano, 2004). It would be exceedingly valuable if appropriate studies could provide zoliflodacin PK data for the urogenital and extragenital infection sites, particularly in the pharynx. In fact, such PK data is lacking not only for potentially novel therapeutics but also for antimicrobials currently used for the treatment of gonorrhea, highlighting the urgent need in generating this type of PK data (Kong et al., 2019). Ideally, PK studies should be included in all RCTs for treatment of gonorrhea and other STIs, however, this may not be feasible and/or cost-effective in many studies. Furthermore, significantly enhanced understanding of pharyngeal gonorrhea and where and how to measure the relevant PK parameters of therapeutic antimicrobials in gonorrhea infection sites, especially in the pharynx, is urgently needed (Kong et al., 2019). Finally, inter-patient variance in PK parameters for zoliflodacin and other gonorrhea therapeutic antimicrobials from population modeling and employing these data in Monte Carlo simulations for target attainment is additionally imperative (Drusano et al., 2001; Drusano, 2004).

The ongoing international phase 3 RCT (ClinicalTrials.gov identifier NCT03959527) is comparing a zoliflodacin 3 g single oral dose to ceftriaxone plus azithromycin dual therapy for treatment of uncomplicated gonorrhea. This study will provide evidence on whether this single dose of oral zoliflodacin, is non-inferior to the globally recognised dual comparator but is not designed to address the suitability of the dose for infections caused by strains with emerging zoliflodacin resistance regardless of body site.

In conclusion, by examining the pharmacodynamics of zoliflodacin against one clinical zoliflodacin-susceptible *N. gonorrhoeae* strain with the pre-existing zoliflodacin-target GyrB S467N substitution in our dynamic HFIM for gonorrhea, we demonstrated that the rare *N. gonorrhoeae* clinical strains with a GyrB S467N substitution are predisposed to develop zoliflodacin resistance and require treatment with zoliflodacin ≥3 g. In the HFIM, zoliflodacin-resistant mutants (with an additional GyrB substitution, i.e., D429N) were selected using zoliflodacin single oral doses of 0.5–2 g. These selected zoliflodacin-resistant mutants (containing GyrB S467N and D429N substitutions) were not eradicated at any of the single- or multiple-dose regimens of zoliflodacin studied in the HFIM. A rapid point-of-care test simultaneously detecting *N. gonorrhoeae* and *gyrB* mutations causing or predisposing to zoliflodacin resistance may be valuable, i.e., for antimicrobial stewardship to avoid zoliflodacin treatment of patients without *N. gonorrhoeae* or with *N. gonorrhoeae* having relevant *gyrB* mutations. However, for this a clinical study evaluating this type of approach would be needed. It is additionally imperative to continue to survey zoliflodacin susceptibility phenotypically as well as genetically, i.e., with emphasis on mutations in *gyrB* or other genes that are verified to cause or predispose to zoliflodacin resistance. Finally, pharmacokinetic data for zoliflodacin (and other gonorrhea therapeutic antimicrobials) in urogenital and extragenital human infection sites, particularly in the pharynx, would be valuable.

Future use of zoliflodacin will require an additional evidence-base to support interventions such as dose adjustments, dual antimicrobial therapy to potentially enhance the bacterial eradication, prevent the emergence and or spread of resistance, and possibly also cure additional STIs.

## Data Availability

The datasets presented in this study can be found in online repositories. The names of the repository/repositories and accession number(s) can be found below: National Center for Biotechnology Information (NCBI) BioProject database under accession numbers PRJEB50904 and ERP135503.

## Supplementary Material

Supplementary Figure 1 Schematic flow chart summarizing the main experiments of the zoliflodacin-susceptible *Neisseria gonorrhoeae* SE600/18 parent strain (with a GyrB S467N mutation) and the zoliflodacin-resistant *N. gonorrhoeae* SE600/18-D429N mutant (with GyrB S467N plus GyrB D429N selected in the Hollow Fiber Infection Model (HFIM)).

Supplementary Figure 2 Predicted-Observed regressions for zoliflodacin concentrations, total *Neisseria* burden and resistant bacterial burden, respectively for the pre-Bayesian regression **(A–C)** and for the Bayesian regressions **(D–F)** for the clinical zoliflodacin-susceptible *Neisseria gonorrhoeae* SE600/18 strain with a GyrB S467N amino acid substitution.
