# Supplementary figures and images for: Pharmacodynamic Evaluation of Zoliflodacin Treatment of Neisseria gonorrhoeae Strains With Amino Acid Substitutions in the Zoliflodacin Target GyrB Using a Dynamic Hollow Fiber Infection Model
Source: Front Pharmacol. 2022 Apr 14;13:874176. doi: 10.3389/fphar.2022.874176 (PMC9046595; doi:10.3389/fphar.2022.874176)

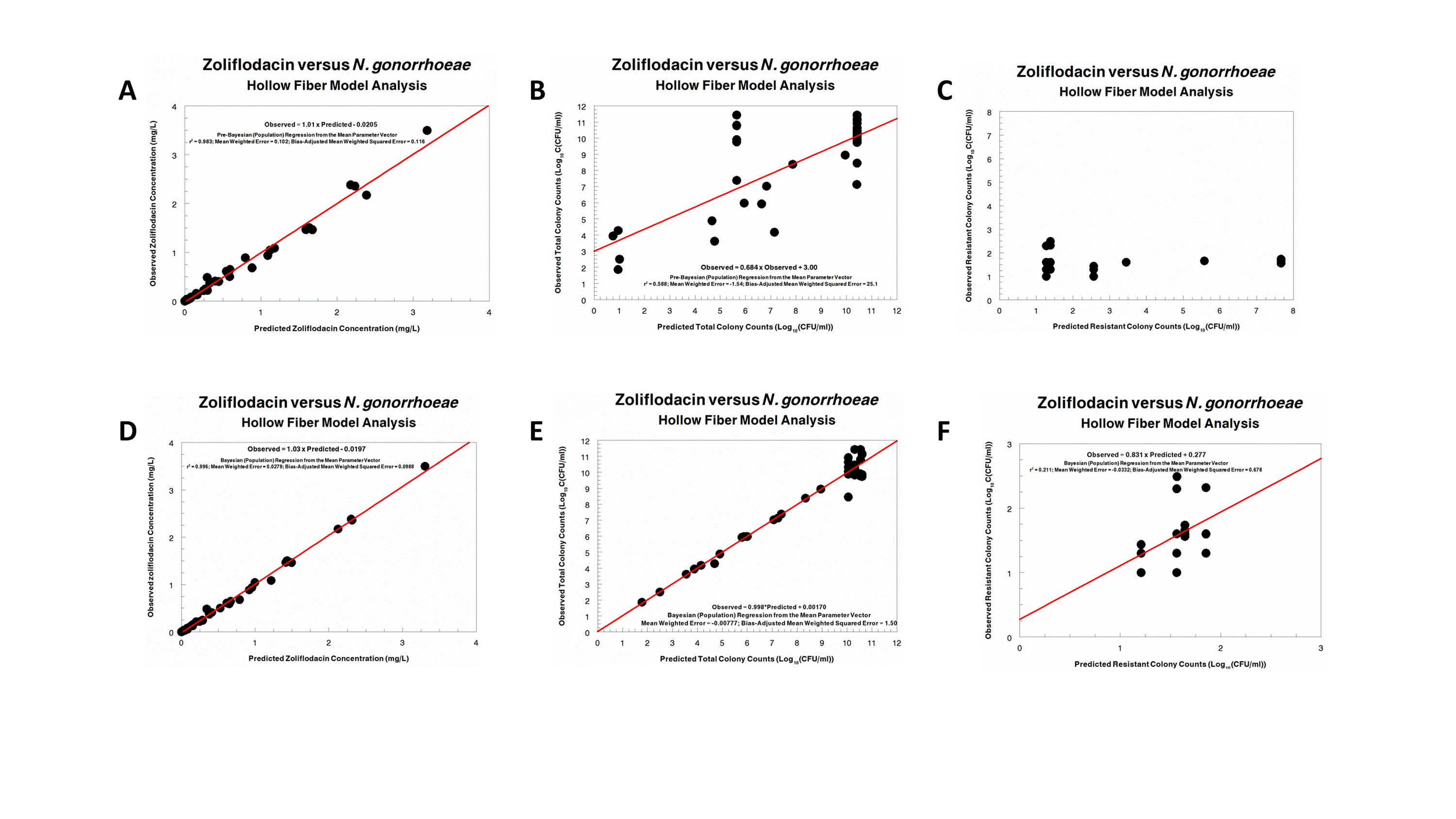

Supplement: Supplementary file 1 [file Image2.tif]

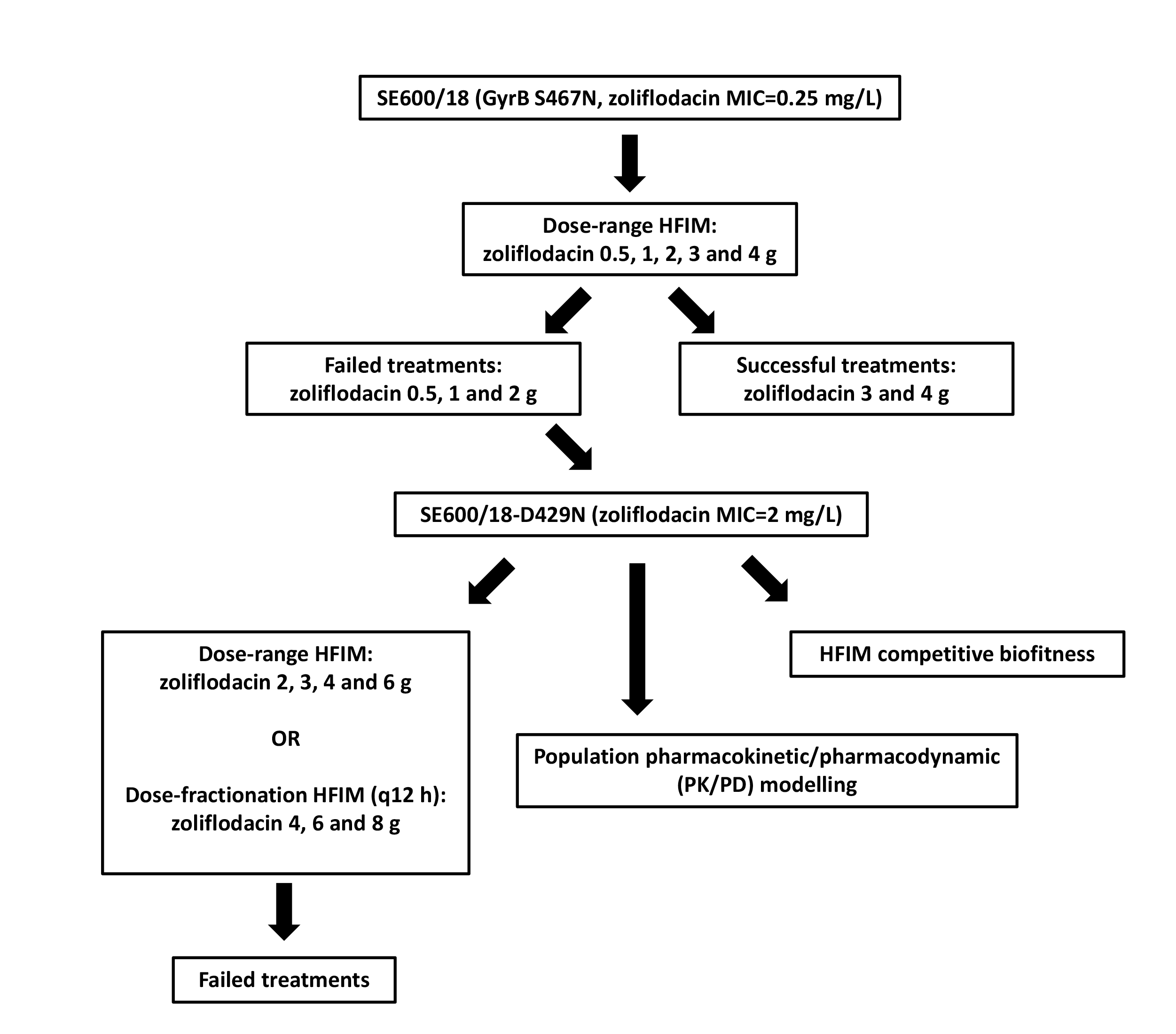

Supplement: Supplementary file 2 [file Image1.tif]
